# A novel multifactorial hamstring screening protocol: association with hamstring muscle injuries in professional football (soccer) – a prospective cohort study

**DOI:** 10.5114/biolsport.2022.112084

**Published:** 2021-12-30

**Authors:** Johan Lahti, Jurdan Mendiguchia, Pascal Edouard, Jean-Benoit Morin

**Affiliations:** 1 Université Cote d’Azur, LAMHESS, Nice, France; 2 Department of Physical Therapy, ZENTRUM Rehab and Performance Center, Barañain, Spain; 3 Inter-university Laboratory of Human Movement Science (LIBM EA 7424), University of Lyon, University Jean Monnet, F-42023. Saint Etienne, France; 4 Department of Clinical and Exercise Physiology, Sports Medicine Unit, University Hospital of Saint-Etienne, Faculty of Medicine, Saint-Etienne, France; 5 Sports Performance Research Institute New Zealand (SPRINZ), Auckland University of Technology, Auckland, New Zealand

**Keywords:** Soccer, Injury prevention, Sprinting, Risk factors

## Abstract

The aim of this pilot study was to analyze the potential association of a novel multifactorial hamstring screening protocol with the occurrence of hamstring muscle injuries (HMI) in professional football. 161 professional male football players participated in this study (age: 24.6 ± 5.36 years; body-height: 180 ± 7.07 cm; body-mass: 77.2 ± 7.70 kg). During the pre- and mid-season, players performed a screening protocol consisting of 11 tests aimed to evaluate their performance in regards to four main musculoskeletal categories: posterior chain strength, sprint mechanical output, lumbopelvic control and range of motion. Univariable cox regression analysis showed no significant association between the isolated test results and new HMI occurrence during the season (n = 17) (p > 0.05). When including injuries that took place between the pre- and mid-season screenings (~90 days), maximal theoretical horizontal force (F0) was significantly associated with higher HMI risk between pre- and mid-season evaluations (n = 14, hazard ratio; 4.02 (CI95% 1.08 to 15.0, p = 0.04). This study identified that 1) no single screening test was sufficient to identify players at risk of HMI within the entire season, while 2) low F0 was associated with increased risk of HMI when occurring closer to the moment of screening. The present results support the potential relevance of additionally including frequent F0 testing for HMI risk reduction management. Replication studies are needed in larger cohorts for more accurate interpretations on “univariable and multivariable levels levels. Finally, future studies should explore whether improving F0 is relevant within a multifactorial HMI risk reduction approach.

## INTRODUCTION

Hamstring muscle injury (HMI) occurrence represents one of the largest injury dilemmas in professional football, ranging between 12–15% of all football-related injuries [1, 2]. This has been shown to compromise team success, caused by both the amount of absence time of the injured players as well as a tendency towards declined performance capacity when returning to sport [3, 4]. Large efforts have been made within prospective research to evaluate and improve awareness of associated intrinsic risk factors [5]. These efforts aim to improve the accuracy of injury risk reduction interventions and player load management [6]. It is generally agreed that HMI risk in football depends on multiple potentially modifiable risk factors [5, 7]. Therefore, hamstring screening protocols should also be multifactorial to better meet the multifactorial nature of the injury and to improve the individualization of interventions.

Musculoskeletal screening protocols (and their included mode of analysis) that accurately predict players at risk for HMI are still in their infancy [6, 8]. This means that it is difficult for contemporary clinicians to make accurate risk management judgment calls based on screening results [6]. Therefore, screening protocols that simultaneously test for performance and HMI risk outcomes may be considered more useful for contemporary clinicians [9]. In this manner, the risk of wasting the time of players with false positive results is reduced. Furthermore, screening protocols should be time- and cost-efficient, as this increases the probability of frequent and widely spread use. More frequent testing may also be essential to the screening information quality, as studies have shown screening results can fluctuate substantially during the season [10, 11]. Thus, organizing additional screening opportunities after the preseason may, in turn, better reflect the current status of the player at the time of injury during the season [5, 12, 13].

Recently, a novel musculoskeletal hamstring screening protocol for football (Football Hamstring Screening: FHS) was introduced [9]. This multifactorial protocol aims to provide clinicians with a cost- and time-efficient alternative for HMI risk management from both a risk reduction and performance perspective [9]. Specifically, the protocol’s 11 tests last ~30 minutes per player, with the total test device budget of ~3000 USD. Furthermore, the an intra-rater study has shown initial promise for the protocol to be reliable within a football cohort [14]. Based on previous literature and anecdotes from experienced practitioners, the FHS has been divided into four screening categories that are considered important in football, including a total of 11 tests. The respective four categories are (1) posterior chain strength, (2) lumbopelvic control, (3) range of motion, and (4) sprint mechanical output [9]. There is now a need to explore the efficacy of the newly introduced hamstring screening protocol for football to identify players at risk for HMI.

Therefore, the primary aim of this pilot study was to analyze the potential association of each of the four components of this screening protocol, evaluated by means of 11 separate tests, with the occurrence of HMI. The second aim was to determine whether screening results change during the season.

## MATERIALS AND METHODS

### Study design and overall procedure

We conducted a single-season prospective cohort pilot study among Finnish professional football players. Screening tests were conducted within this cohort during the end of pre-season (March 2019) and the mid-season (June 2019) periods. Prospective data collection as regards sport exposure and injury occurrence were collected throughout the entire season, from April to October 2019. The study was approved by the Saint-Etienne University Hospital Ethics Committee (Request number: IORG0007394; Record number IRBN322016/CHUSTE).

### Population

We recruited 161 football players from nine teams using convenience sampling (age: 24.6 ± 5.36 years; body-height: 180 ± 7.07 cm; body-mass: 77.2 ± 7.70 kg), with one recruited from the primary league in France (Ligue 1), and eight from the premier Finnish football division (Veikkausliiga). The objectives, procedures, and risks of the study were explained to the coaching staff and players through verbal discussions, documentation, and oral presentations. Inclusion criteria included completing all screening tests during the preseason measurements, playing the entire in-season in the same team, and injury and exposure data being collected according to the study guidelines. Exclusion criteria included having ongoing rehabilitation and being a goalkeeper, since the respective playing position carries a low HMI risk [15]. All participating players provided written informed consent prior to study participation.

### Data collection

All included football players completed 11 tests included in a hamstring screening protocol during the pre- and mid-season. We performed two screening tests sessions to try to account for seasonal fluctuations in physical scores [10, 11]. All tests were conducted in each team’s training environment (clinic and on-field). The FHS is presented in the Table 1, with details of its four components and 11 screening tests, as well as the intrinsic risk factors, the musculoskeletal elements, the assessed variables, and the corresponding experimental equipment used in testing. All measurements were carried out in each teams testing quarters by the same experienced practitioner (JL). At the start of the season (i.e., during the preseason testing), previous HMI within the last two seasons, playing position, age, and basic anthropometrical information (height, bodymass), were recorded for all players through questionnaires. Body-mass data were updated during the mid-season testing. Injury history was confirmed by the team physiotherapist and anthropometric information was measured during the first day of screening testing.

**TABLE 1:** Football Hamstring Screening protocol with its four components, including the 11 screening tests and their respective methods and equipment used.

Component of the screening protocol	Anatomical elements/property	Assessed variable	Experimental equipment
Lumbo-pelvic control	Pelvic movement in normal gait Sprint kinematics (“Kick-back mechanism”)*	Peak pelvic anterior/posterior tilt and obliquity during walking (10-m) Thigh angle during touchdown and toe-off in maximal upright sprinting*	Gyroscope sensor [21] Slow motion camera [14]
Posterior chain strength (+ asymmetry)	Hip extensor isolative strength Knee flexors isolative strength	Isometric force at 0^o^ of hip ext. and 95–100^o^ of knee flexion (N · kg^-1^) Isometric force at 0^o^ of hip ext. and 25–30^o^ of knee flexion (N · kg^-1^)	Hand-held dynamometer Microfet II [24]
Range of motion (+ asymmetry)	Hamstrings extensibility Hamstrings extensibility in combination with hip flexors	Thigh angle during active straight leg raise (ASLR) Active knee extension with opposite thigh passive angle (Jurdan test)	Goniometer records app [19]
Sprint mechanical output	Dynamic posterior chain strength during maximal sprint acceleration	Maximal horizontal force (F*0*) during 2 × 30-m sprints (N · kg^-1^)	Stalker ATS II radar [26]

Note: * Sprint kinematic testing (Lumbopelvic control) was tested at the same time as sprint mechanical output testing

The FHS consists of 11 screening tests within the following four categories; posterior chain strength, sprint mechanical output, lumbopelvic control, and range of motion. Each of the four categories are further divided into clinical tests and field tests. Familiarization was conducted separately for specific tests that were considered to have a learning curve (listed in Table 1). The FHS testing battery was designed to be efficient and mobile, taking roughly 30 min to conduct per participant, with the clinical tests requiring no general warm-up. A total of nine clinical tests were performed in the following order; two range of motion and asymmetry tests, two posterior chain strength and asymmetry tests, and one lumbopelvic control test. The field tests included sprint mechanical output testing that was combined with the second lumbopelvic control test (The “Kick-back” test during sprinting). These tests lasted seven minutes per participant and required a standardized sprint-specific 15-minute warm-up. Sessions were planned according to the teams’ schedules so that there were ideally no matches 72 h before the clinical tests and 96 h before the sprinting tests. Two measurements were obtained and averaged per variable.

All tests are described in more detail in previous work [9]. The test-retest intra-rater reliability of the protocol has been assessed by the same research group and performed by the same clinician (JL). In all 11 tests, inter-day intraclass correlation coefficients (ICC_3,2_) ranged from moderate-to-excellent (0.72 – 0.99), and relative reliability (minimal detectable change) ranged between 6.63 – 21.5% [14].

**FIG. 1:**
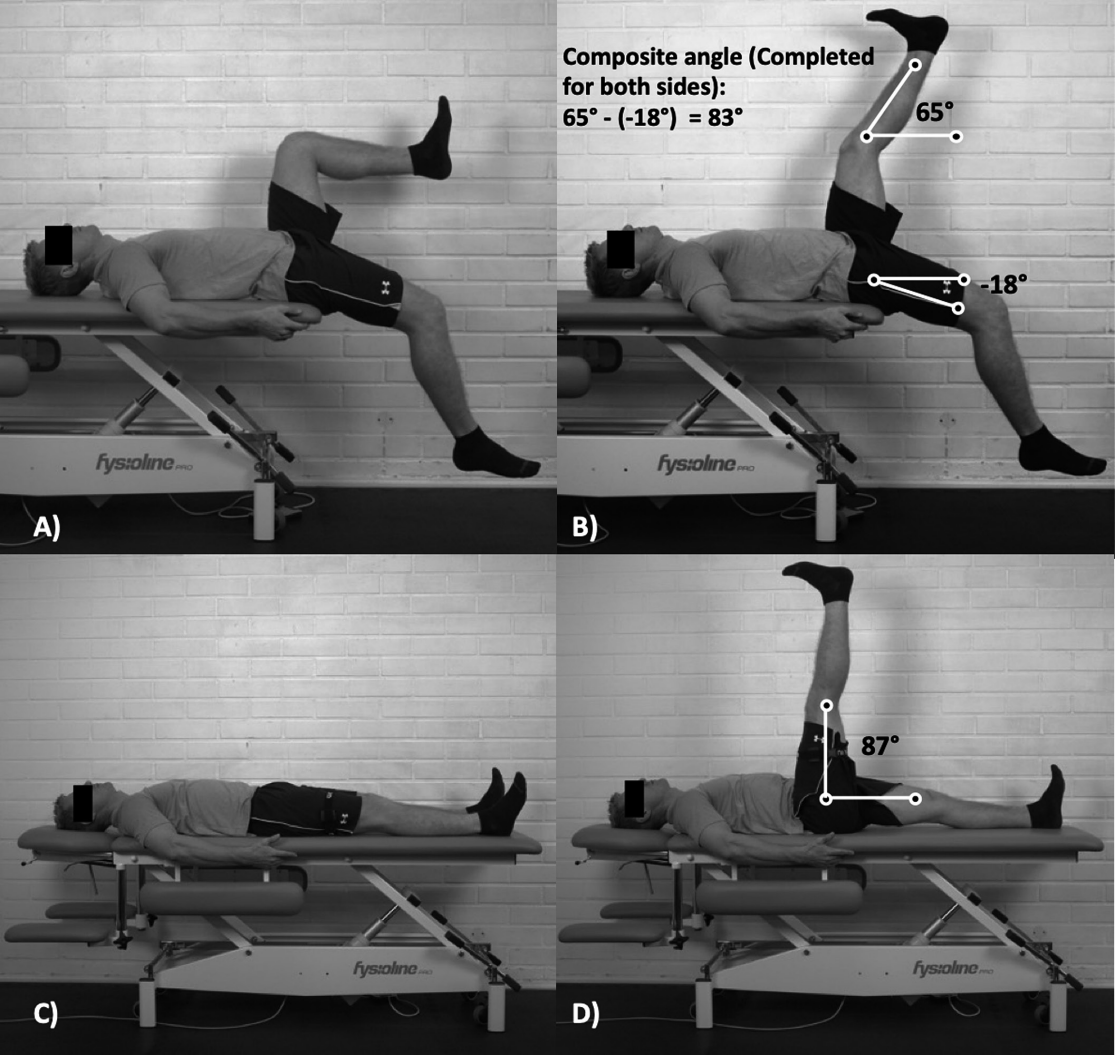
Range of motion tests. The novel Jurdan test (A, B) is based on a composite score from two measurements; the active maximal knee extension angle and the opposite legs passive hip flexion angle. The ASLR test (C, D) is based on the maximal active straight leg hip flexion angle. Asymmetries are calculated from both tests. Therefore, a total of four tests are analysed within the range of motion category. Figure used with permission from Lahti et al. [14].

Two range of motion tests were performed, both assessing between limb asymmetry (Figure 1). The first test was the novel “Jurdan test” followed by the active straight leg raise (ASLR) test. The Jurdan test has never been used in previous injury risk research (Figure 1, A & B). The test aims to consider the influence of the muscles of the lumbopelvic region on hamstring extensibility, which has long been proposed to contribute to hamstring strain injuries [16]. Most notably, the iliopsoas has been shown to have the largest magnitude of influence on the hamstrings length during sprinting. Thus, the Jurdan test can be considered a combination of the active knee extension test [12] and the modified Thomas test that is commonly used to assess iliopsoas [17]. As demonstrated in Figure 1, the Jurdan test result is defined as the difference between the shin angle of the actively lengthened leg and the thigh angle of the opposite leg. The ASLR test (Figure 1, C & D) measures the active thigh angle from a straight leg raise and is considered to have good reliability, sensitivity, and specificity [18]. Two range of motion tests were included in the protocol to potentially control for different strain-related injury scenarios. The Jurdan test is proposed to be more related to sprinting, while the ASLR more to overstretching actions (e.g., slide-tackling, and high kicks). According to our data, the test outcomes were correlated by r = 0.56. This means that although the results are partly related, they also show clear independence. For both tests, limb angles were measured manually using a validated digital goniometer (Goniometer records, Indian Orthopedic Research Group) [19]. Between-limb asymmetry was calculated using the following formula: (100/maximum value)*(minimum value)*-1*100) as proposed by Bishop et al. [20].

**FIG. 2:**
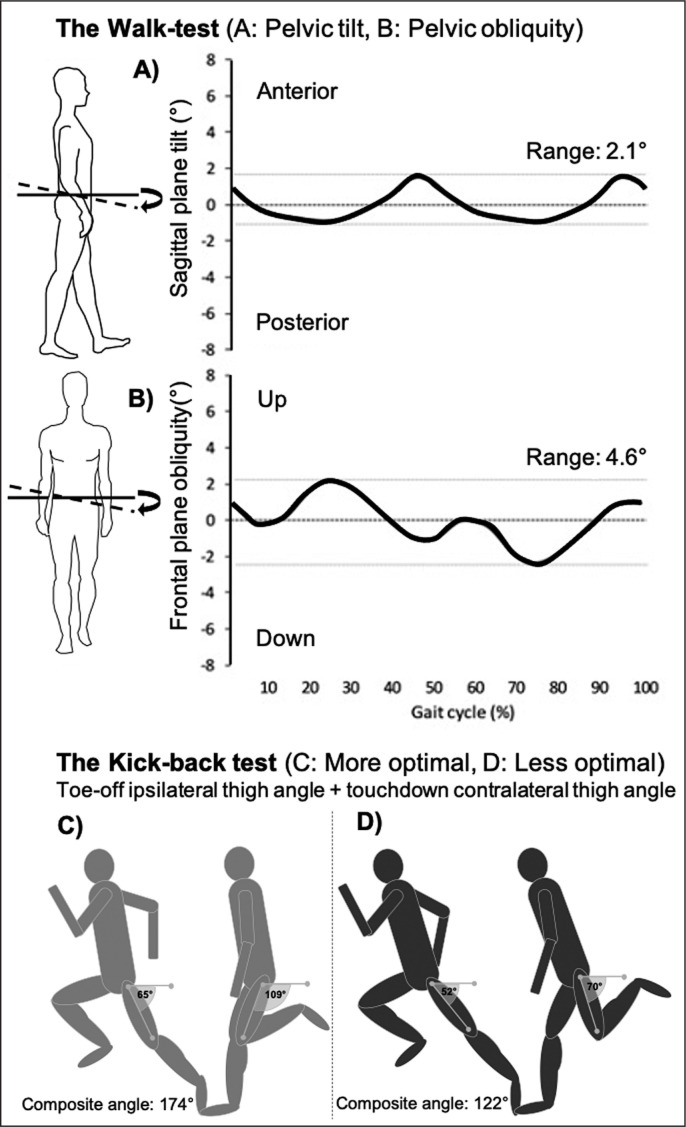
Lumbopelvic control tests. The Walk-test (A, B) is based on a composite score of the sagittal and frontal plane kinematic range of the pelvis during walking. The novel Kick-back test (C, D) is based on a composite score from two measurements; the ipsilateral thigh angle during toe-off and the contralateral thigh angle touchdown. Figure used with permission from Lahti et al. [14].

Two lumbopelvic control tests were performed, including the “Walk test” followed by the sprint kinematic “Kick-back” test (Figure 2). The latter test was completed in combination with sprint mechanical output testing. The Walk test (Figure 2, A & B), consisted of measuring the peak dynamic sagittal and frontal plane pelvic movement in normal gait by placing a gyroscope sensor on the S1/L5 junction (LetSense Group, Castel Maggiore, Italy) [21]. The second lumbopelvic control test “Kick-back” is new to the literature and aims to indirectly assess lumbopelvic control by measuring the thighs interaction in upright sprinting (Figure 2, C & D). This thigh interaction may be related to the degree of anterior pelvic tilt in sprinting [22], which has been associated with increased risk of HMI [23]. The thigh angle was analyzed with open access video analysis software (Kinovea, v.0.8.15) from two adjacent steps within two sprints using a high framerate slow motion camera (Iphone6, Apple Inc, Cupertino, Ca).

**FIG. 3:**
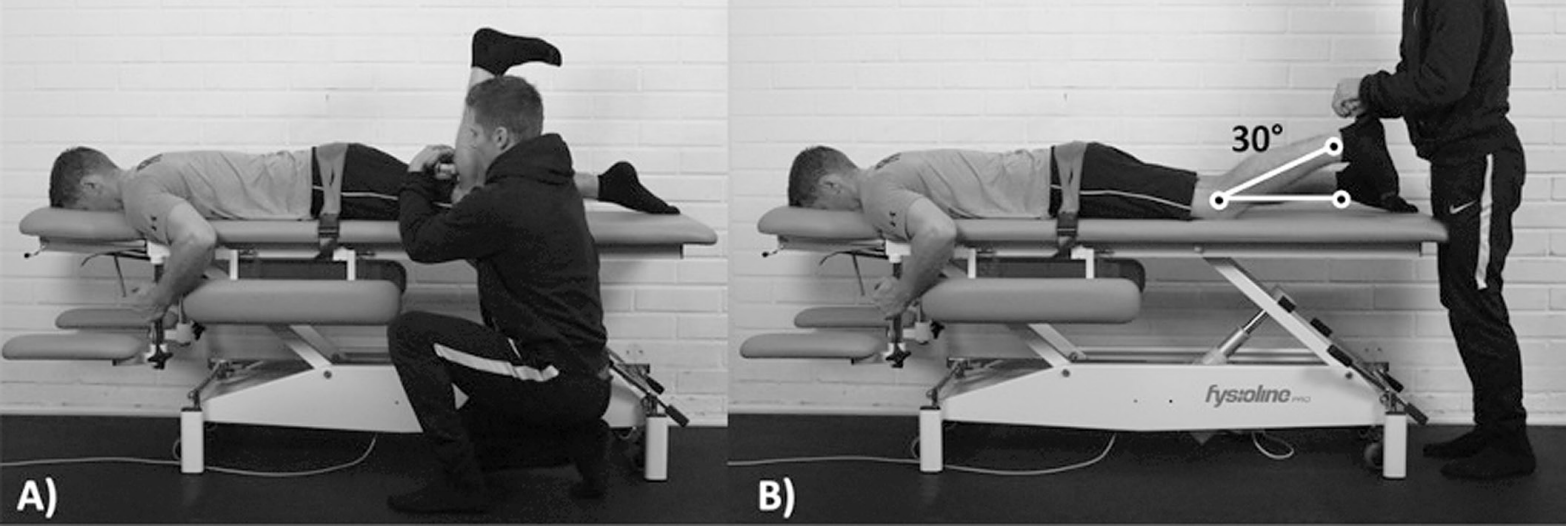
Posterior chain strength tests. The hip extensor strength test (A) and the knee flexor strength test (B) measure strength via a maximal voluntary isometric contraction using manual dynamometry. Asymmetries are calculated in both tests. Therefore, a total of four tests are analysed within the posterior chain strength category. Figure used with permission from Lahti et al. [14].

Limb strength and asymmetry was investigated using two isometric posterior chain strength tests (Figure 3). The first one consisted of an isometric knee flexor strength test, while the second one was a hip extensor strength test; both tested using a hand-held dynamometer (microFET IITM, Hoggan health industries, Draper, UT, USA). Both test positions have been reported to be reliable in previous literature [24, 25]. Between-limb asymmetry was calculated with the same method as mentioned in the range of motion section [20].

**FIG. 4:**
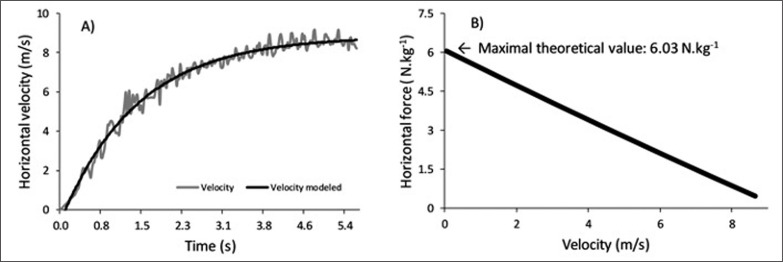
Sprint mechanical output. Raw velocity data from a radar gun is fitted with an exponential function (A). Thereafter, a sprint force-velocity profile is created (B). The variable of interest is the extrapolated maximal theoretical horizontal force value (In figure B it is 6.03 N · kg^-1^). Figure used with permission from Lahti et al. [14].

Sprint mechanical output was assessed by measuring theoretical maximal horizontal force (F*0*) from two 30-m maximal sprints (Figure 4). Participants were granted three minutes of rest in between both maximal trials. Measurements were performed after a structured warm-up, including approximately 5-min jogging, 5-min dynamic stretching, 1–2 minutes of sprint drills, and 2 × 10 m and 2 × 30 m sprints with increasing intensity, and with small variations according to teams. To standardize tests and improve reliability, all sprints were completed outdoors on synthetic turf in calm weather (wind speed < 2.5 m · s^-1^). To improve the reliability of the 2D sprint kinematics assessment (investigating lumbo-pelvic control), participants were instructed to run along the field line. F*0* was computed using a validated field method measured with a radar device (Stalker ATS Pro II, Applied Concepts, TX, USA) [26]. Briefly, inverse dynamics is used to calculate F*0* using the time-motion data of the center-of-mass. An exponential function is fitted on the raw velocity–time data. The instantaneous data is combined with system mass (body-mass) and aerodynamic friction to compute the net horizontal antero-posterior ground reaction force. Thereafter, individual linear sprint force–velocity profiles are extrapolated to specify F*0*.

### Sport exposure and injury data collection

Sport exposure was defined as average weekly training volume (expressed in hours and at group level) and match time (expressed in playing hours at individual level) and collected by the team’s strength and conditioning coach.

Injury was defined as any musculoskeletal lesion (sustained through trauma or overuse) occurring during a scheduled training session or official match and causing absence from the next training session or match [27]. Injury data were collected and registered by each team’s physiotherapist using a standardized report form including various information (e.g., date, circumstances (match/training), injury location, type, cause, and date of return to play). The primary outcome of this study was the HMI occurrence, which is defined as an injury located at the posterior side of the thigh and involving muscle tissue [28]. Hamstring injuries described as cramping/spasm by the physiotherapist (in absence of an actual structural lesion/tear), were included as muscle injuries in our work. This was due to the absence from playing time related to these functional hamstring injuries. The diagnosis was made clinically and confirmed by ultrasound or MRI. To avoid any rehabilitative attempts of correcting functional asymmetries detected at the pre-season testing, neither players nor their physiotherapists were informed about the results of the entire screening protocol before the completion of the study.

### Data analysis

Descriptive analysis was performed for sport exposure, player characteristics, injuries, and screening test results. Categorical variables were reported as frequencies with percentage distributions and continuous variables were reported as the mean with standard deviations (± SD). Injuries were reported as total number of HMI, HMI incidence (per 1000 hours of training, match and total football exposure) and burden of HMI (days lost due to HMI per 1000 hours of exposure).

Then, the population was divided into non-injured (i.e., players who did not sustain any HMI during the entire season) and injured groups (i.e., players who sustained an HMI during the whole season). An independent t-test was used to assess the potential differences between non-injured and injured groups. In addition, a paired t-test was used to test for possible differences between pre- and mid-season testing among all players who performed the two screening tests. For both of the mean comparison approaches described above, effect sizes were calculated, which were subsequently qualitatively interpreted as small (≥ 0.2), moderate (≥ 0.6), large (≥ 1.2), very large (≥ 2.0), and nearly perfect (≥ 4.0) effects [29].

Univariable Cox proportional hazards regression (or Cox regression) with a ‘time-to-event’ approach was adopted to analyze the association between tests scores and HMI occurrence. Time to the first event was analyzed using a time scale consisting of total hours of football exposure (i.e., training and matches). The cox regression was adjusted for team, age, height, body mass, and history of previous HMI during the last two seasons. The hazard ratio (HR) with a 95% confidence interval (95% CI) are presented for each variable. The assumption that the HR was constant over time was tested. Two models were used, with the first model using the screening tests values at the start of the season and new HMI occurring during the entire season as the outcome, similar to previous literature [12, 30]. The second model aimed to account for changes in screening variables during the season [10, 11], and thus included HMI occurring between the pre- and mid-season (shorter period), leading to a cut-off point of 90 days (mean time between the pre- and mid-season screening sessions; 94 days, CI95%: 92.2–97.2). The researcher who performed the cox regression analyses (PE) was independent of football groups and did not conduct the measurements.

Significance was accepted at p < 0.05. Analyses were performed using Excel (Office, Microsoft®, 2017) and R (version 3.6.3., © Copyright 2016 The Foundation for Statistical Computing (Comprehensive R Archive Network, http://www.R-project.org)).

## RESULTS

### Population and exposure

Within the sample of 161 potential football players, one entire team (18 players) was excluded due to incorrect injury data collection, another team (25 players) was excluded due to preseason scheduling issues that led to not completing clinical tests, 16 players had ongoing rehabilitation (all teams), five players switched teams during the season, and two players did not complete sprint testing. Consequently, the final sample considered for data and statistical analyses consisted of 95 professional football players (age: 24.9 ± 5.33 years; body-height: 181 ± 7.11 cm; body-mass: 77.0 ± 7.39 kg), all competing in the Finnish premier league.

This sample’s total exposure time throughout the season of interest was 26479 hours (24822 training hours and 1657 match hours). The mean training session exposure and match exposure per player were 264 ± 39.1 hours and 21.5 ± 1.74 hours, respectively, during the 28 weeks of official competition. 73% of the 95 players who completed the pre-season screening session also completed the mid-season screening session (n = 69). The other players were not available due to ongoing injuries (n = 14) and scheduling issues (international matches, n = 9, misunderstanding of testing timetable, n = 2).

### Hamstring injuries

There were 17 new HMI, including three that occurred after mid-season screening session. The majority of HMI occurred during sprinting (70%) and involved the Biceps Femoris Long Head muscle (80%). Incidence of HMI was 8.50 injuries per 1000 match hours and 0.47 injuries per 1000 training hours (total injury incidence: 0.76 per 1000 hours). HMI burden was 14.1 days per 1000 hours of football exposure. More information on HMI occurrence is presented in Table 2.

**TABLE 2:** Number, prevalence, incidence, and nature of all HMI

**HMI occurrence (n,% of the population)**	
During season	20 (24)
New injuries	17 (18)
Reinjuries	3 (3)
Previous injuries (last two seasons,%)	23 (24)
**HMI Injury incidence per 1000 h (CI95%)**	
Total injury incidence	0.76 (0.45–1.22)
Injury incidence, training	0.47 (0.31–0.79)
Injury incidence, match	8.50 (5.21–13.7)
**Injury severity (n,% of HMI)**	
Mild (4–7 days)	4 (20)
Moderate (8–28 days)	13 (65)
Severe (> 28 days)	3 (15)
**Position (n,% of new HMI)**	
Defender	5 (29)
Midfielder	6 (35)
Forward	6 (35)
**Circumstances** (n,% of HMI)	
Match	11 (55)
Training	9 (45)
**Mechanisms (n,%)**	
Sprinting	14 (70)
Change of direction	3 (15)
Slide tackle	2 (10)
Unknown	1 (5)
**HMI time-loss and injury burden (CI95%)**	
Days of absence/injury	18.5 (14.0 – 22.9)
Injury burden (1000 h of football exposure)*	14.1 (6.30 – 27.9)

* : Total HMI injury incidence × days of absence from HMI

### Changes in screening results during the season

Screening results from both pre- and mid-season session are presented in Table 3. From the total of 69 players completing pre- and mid-season screening session, there was a significant increase in knee flexor strength (3.77 vs 3.99 N · kg^-1^, p < 0.0001, ES: 0.35) and maximal theoretical horizontal force (7.63 vs. 7.84 N · kg^-1^, p = 0.004, ES: 0.35), and a significant decrease in ASLR asymmetry (6.75 vs 4.36, p = 0.0001, ES: -0.60) when comparing the pre- and the mid-season results.

**TABLE 3:** Player characteristics and screening tests results from the Football Hamstring protocol

Categories	Variables	Comparison between non-injured and injured groups (preseason)				Comparison between players completing both pre- and mid-season testing			
		Non-injured (n = 78, CI95%)	Injured (n = 17, CI95%)	*p* value	Effect size	Pre-season testing (n = 69, CI95%)	Mid-season testing (n = 69, CI95%)	*p* value	Effect size
Player information	Age	24.6 (23.4; 25.8)	26.4 (24.3; 28.4)	24.95 (23.8; 26.1)					
	Weight	1.80 (1.79; 1.82)	1.82 (1.78; 1.84)	1.81 (1.79; 1.83)					
	Height	77.0 (75.3; 78.7)	76.9 (73.7; 80.1)	77.11 (75.5; 78.7)					
	Previous injury, n (%)	11 (14.0)	6 (35.2)	12 (100)					
Lumbo-pelvic control	Walk test (^o^)	8.88 (8.35; 9.42)	8.85 (7.67; 10.0)	0.96	-0.01	8.79 (8.26; 9.32)	8.57 (8.12; 9.0)	0.48	-0.10
	Kick-back test (^o^)	146 (144; 149)	143 (137; 149)	0.24	-0.31	146 (143; 148)	0.62	-0.01
Posterior chain strength	Knee flexor strength (N · kg^-1^)	3.78 (3.64; 3.93)	3.75 (3.46; 4.03)	0.83	-0.06	3.77 (3.63; 3.90)	3.99 (3.84; 4.13)	< 0.0001*	0.35
	Knee flexor strength asymmetry (%)	6.40 (5.27; 7.53)	8.11 (5.42; 10.8)	0.22	0.32	6.64 (5.55; 7.74)	7.56 (6.00; 9.10)	0.26	0.15
	Hip extensor strength (N · kg^-1^)	4.16 (3.97; 4.35)	4.35 (4.05; 4.66)	0.38	0.26	4.26 (4.08; 4.44)	4.42 (4.24; 4.61)	0.07	0.19
	Hip extensor strength asymmetry (%)	7.46 (6.20; 8.72)	6.48 (4.22; 8.74)	0.51	-0.19	8.05 (6.57; 9.52)	0.29	0.06
Range of motion	ASLR (^o^)	87.5 (85.7; 89.3)	86.4 (82.3; 90.5)	0.64	-0.12	88.7 (86.8; 90.5)	88.0 (86.2; 89.9)	0.28	-0.07
	ASLR asymmetry (%)	6.18 (5.20; 7.17)	7.04 (4.42; 9.61)	0.51	0.17	6.75 (5.72; 7.78)	4.36 (3.67; 5.05)	0.0001*	-0.60
	Jurdan test (^o^)	79.1 (76.7; 81.5)	77.0 (70.4; 83.7)	0.50	-0.17	79.40 (76.7; 82.1)	80.2 (77.2; 82.7)	0.44	0.07
	Jurdan test asymmetry (%)	7.53 (6.13; 8.94)	7.01 (4.36; 9.71)	0.77	-0.08	7.18 (5.83; 8.54)	8.39 (6.84; 9.95)	0.26	0.18
Sprint mechanical output	Maximal theoretical horizontal force (N · kg^-1^)	7.67 (7.54; 7.80)	7.46 (7.18; 7.74)	0.22	-0.35	7.63 (7.50; 7.76)	0.004*	0.35

^o^: degrees, ASLR: Active straight leg raise, N: Newton, kg: kilogram,

* : p < 0.05

### Association between screening tests and HMI risk

The results from the two univariable models of the cox regression are presented in Table 4. The first cox regression model showed no significant association between any screening test and increased HMI risk, including each HMI occurring during the entire season. In the second model, which accounted for injuries that occurred between pre- and mid-season measurements (therefore only including HMI occurring throughout the first half of the season), lower F*0* was significantly associated with HMI occurrence (hazard ratio [HR], 4.02 (CI95% 1.08 to 15.0, p = 0.04) (Table 4). No other variable changes between pre- and mid-season testing reached significance in function of HMI occurrence. However, a trend was established for higher pre-season hip extensor strength being associated with increased risk of HMI (Table 4).

**TABLE 4:** Cox regression results

Cox regression for all HMI during season (n = 17)					
Categories	Tests	Univariable analysis			
		HR	95%CI	P Value	TI (*p*-value)
Lumbo-pelvic control	Walk test	0.97	(0.78 to 1.20)	0.78	0.28
	Kick-back test	0.97	(0.92 to 1.02)	0.26	0.31
Posterior chain strength	Knee flexor strength	1.46	(0.58 to 3.65)	0.42	0.20
	Knee flexor strength asymmetry	1.04	(0.93 to 1.16)	0.53	0.27
	Hip extensor strength	1.93	(0.94 to 3.95)	0.07	0.24
	Hip extensor strength asymmetry	0.97	(0.87 to 1.08)	0.56	0.22
Range of motion	ASLR	0.97	(0.91 to 1.04)	0.43	0.27
	ASLR asymmetry	1.07	(0.96 to 1.19)	0.25	0.25
	Jurdan test	0.99	(0.94 to 1.05)	0.83	0.06
	Jurdan test asymmetry	0.99	(0.91 to 1.08)	0.85	0.27
Sprint mechanical output	Maximal theoretical horizontal force (FO)	2.98	(0.98 to 9.07)	0.06	0.13
**Cox regression for all HMI between pre- and mid-seasons screening session (within 90 days) (n = 14)**					
Lumbo-pelvic control	Walk test	0.88	(0.68 to 1.12)	0.29	0.06
	Kick-back test	0.99	(0.94 to 1.05)	0.69	0.05
Posterior chain strength	Knee flexor strength	1.45	(0.52 to 4.06)	0.48	0.07
	Knee flexor strength asymmetry	1.05	(0.93 to 1.19)	0.44	0.14
	Hip extensor strength	2.32	(1.00 to 5.37)	0.05	0.16
	Hip extensor strength asymmetry	0.96	(0.85 to 1.09)	0.53	0.11
Range of motion	ASLR	0.97	(0.90 to 1.04)	0.39	0.14
	ASLR asymmetry	1.07	(0.94 to 1.20)	0.31	0.14
	Jurdan test	0.98	(0.93 to 1.04)	0.60	0.05
	Jurdan test asymmetry	0.98	(0.89 to 1.08)	0.66	0.13
Sprint mechanical output	Maximal theoretical horizontal force (FO)	4.02	(1.08 to 15.0)	0.04*	0.09

HMI: Hamstring muscle injury, ASLR: Active straight leg raise,

* : p < 0.05.

## DISCUSSION

The main findings of this study revealed that 1) no screening test in isolation was associated with a new HMI occurring during the entire season, and 2) lower maximal horizontal force production capacity (F*0*) was significantly associated with increased HMI risk when assessing injuries that occurred between the pre- and mid-season testing sessions.

The finding that no screening test in isolation was associated with a new HMI during the entire season was foreseeable based on previous literature [5, 6, 8, 31]. Studies exploring the association between modifiable intrinsic risk factors in isolation and HMI occurrence have shown conflicting results or are limited [5]. The most evident reason is likely due to the difficulty of controlling for the complex nature of injury etiology [32]. Including large samples that have been tested in a multifactorial format is considered of high importance, as it allows for the use of multivariable statistical models [31, 32]. In turn, this may allow answering whether the screening protocol itself is effective, instead of focusing on the potential relevance for specific tests in isolation. However, other considerations are likely also important, such as controlling for changes of tests during the season and increasing the precision of tests [12, 17, 23].

### The potential value of including F0 into screening and monitoring practices

Our pilot study demonstrated that there may be additional strengthrelated outcome measures sensitive to identify players with increased HMI risk, other than the generally proposed strength measures. The hamstrings have been identified as essential protagonists in contributing to the horizontal force component of the ground reaction force vector (i.e. accelerating the center of mass forward) in sprinting [33, 34], which is where most hamstring injuries take place [2, 15]. This premise is supported by a recent modelling study, which was the first study to model muscles contribution to the majority of the sprint acceleration phase [34]. The authors reported that the hamstrings functioned as an essential accelerator through the entire sprint alongside the triceps surae and gluteus medius [34]. The target with hamstring strength testing is to gain insight of the possible load tolerance of the biarticular hamstrings [35]. Thus, tests that assess force output during dynamic actions that emphasize both hip extension and knee flexion mechanical effort could be of interest. Therefore, it was the interest of this study was to test whether measuring horizontal force during sprinting could also indirectly characterize the health status of the hamstrings within their contribution to accelerated run. Specifically, an association of increased risk was found for low levels of F*0* when including injuries between pre- and mid-season screening rounds. The increased accuracy of assessing injuries in closer proximity to testing is supported by previous screening literature [13, 36]. F*0* is a macroscopic variable, which reflects the sum of its parts. Thus, it does not give accurate information on the microscopic role of a single part in the system, such as specific muscle forces. However, the practicality of measuring F*0* and its wider use also as a performance measure may outweigh its limitations when used in a multifactorial testing environment. Furthermore, recent developments in technology allows for reliable *in-situ* quantification of F*0* from football training using global positioning devices [37]. This is a promising development from a testing frequency standpoint and should be further explored as it allows screening practices to evolve into a monitoring context. Moreover, horizontal force appears to be trainable in football [38, 39]. Consequently, there is emerging evidence that the evaluation of hamstring strength, or rather ‘force output’, should consist of multifactorial testing (mostly eccentric strength combined with sprint F*0* testing, based on contemporary evidence) and with frequent scheduling. Despite the difficulty in recruiting professional football athletes, studies with larger sample sizes are needed to confirm this finding and the associated clinical implications/recommendations.

### Accounting for changes in screening results during the season

The limitation of not accounting for changes in screening results during the season in prospective cohort studies assessing HMI risk factors has been discussed in literature [12]. As demonstrated by studies in professional cohorts, clinical and functional test results can substantially change over the course of a competition season [10, 11]. When comparing this study’s pre- and mid-season data, three outcome measures improved significantly with small to moderate effects (ASLR asymmetry, knee flexor relative force, and F*0*). However, some caution is warranted in interpreting these results as these changes can be due to normal weekly fluctuations in testing scores caused by measurement error and or fatigue. To improve interpretation, inter-rater test-retest reliability needs to be explored for the screening tests, preferably also in a setting of professional football players.

We additionally analyzed the changes in tests within each team. There were on average three screening test variables that showed moderate to large effect size changes within five out of seven teams (two teams showed no changes). A total of 16 substantial changes were observed. Only five changes were considered as moving in a clinically negative direction (i.e., less force output, range of motion, and increased asymmetry). When observing the team’s practices, the positive changes were likely largely due to the constant ongoing efforts of reducing the risk of injuries during the season. Furthermore, nearly all in-season test-outcome changes concerned range of motion and force output variables, which were most regularly addressed during the seasonal training planning according to the coaches of the participating teams. They were considered as the most essential for both injury risk management and performance optimization. Therefore, relatively low player performance capacity at the start of the season may partially explain why most injuries occurred near the beginning of the season. This finding has been established in previous research including other cohorts of professional athletes as well [40]. The team coaches speculated that one explanation for the increased HMI risk in the early season is the heavy preseason loading (i.e., the substantial change in athlete loading when comparing off- and preparation season phases). This preparation phase consisted of a combination of practice matches and pre-season tournaments in this cohort, minimizing the time left to spend on injury risk reduction strategies.

The fact that most existing injury-risk identification-related research does not consist of repeated risk factor screening sessions throughout the season is understandable. Pre-season screening protocols can be potentially fatiguing and time-consuming (especially if tests are completed in separate facilities). Furthermore, high sample size pre-requisites and repeated voluminous testing data collection likely require collaboration between multiple research groups to deliver study results with sufficient power [32]. Future studies should aim to account for changes in screening results in even closer intervals or insert continuous monitoring evaluation strategies in their athletic samples. This should include cognitive and emotional data collection next to the commonly adopted clinical and functional musculoskeletal outcome parameters [8].

### Strengths and limitations

The main strength of this pilot study was that it considered the multifactorial etiology of HMI in professional football, investigating the role of lumbo-pelvic control, range of motion, posterior chain strength and sprint mechanical output for the HMI risk, while introducing novel tests. The FHS protocol has been successfully implemented in several professional teams after education to physiotherapy and physical conditioning staffs in an ongoing intervention study [9], which supports its feasibility in real-life scenarios. Another strength was that analyses were adjusted for important confounding factors, including football exposure, body-mass, team, age, and history of previous HMI, or the samples were otherwise homogenous (e.g., sport exposure [an average of one match and 5–6 days of training per week], weather, level of play, and resting periods).

The main limitation of this study is the low final sample size, which hinders clear conclusions. With this in mind, while multivariable models have been advised to be used for multifactorial injuries [32], such a regression model including all 11 tests and confounding factors would have required a much larger sample [31]. However, univariable analysis such as in this study is also considered important, as potential associations help to spot relevance of risk factors [32]. Finally, it should be mentioned that advancements in technology may lead to specific devices used in this study becoming obsolete. Specific methods used in this study for analyzing the raw data can be considered relatively slow (such as the assessment of F*0*, or the Kick-back mechanism). Furthermore, achieving highly accurate associations between the chosen lumbo-pelvic tests (i.e., pelvic kinematics during normal gait or indirect 2D analysis during sprinting) and pelvic kinematics during dynamic football actions are unlikely. Direct measurements of pelvic kinematics during maximal sprinting, or other relevant kinematic and spatiotemporal variables measured during football exposure would allow for less extrapolation of inferences. Additionally, a separate assessment of the sagittal and frontal plane mechanics should be explored. Thus, constant updates in technology will likely allow clinicians to get accurate results faster within the testing categories of interest.

## CONCLUSIONS

This study demonstrated that no single screening test was associated with increased HMI risk in professional football when considering HMI taking place during the entire season. However, when analyzing hamstring injuries that took place throughout the first half of the season when injury incidence was the highest (before mid-season testing (90 days)), lower F*0* was associated with an increased risk of sustaining an HMI. Thus, there may be potential relevance in frequently monitoring F*0* levels during the season in professional football to further improve HMI risk reduction approaches.
